# G-bic: generating synthetic benchmarks for biclustering

**DOI:** 10.1186/s12859-023-05587-4

**Published:** 2023-12-06

**Authors:** Eduardo N. Castanho, João P. Lobo, Rui Henriques, Sara C. Madeira

**Affiliations:** 1 LASIGE, Faculdade de Ciências, Universidade de Lisboa, Campo Grande 016, 1749-016 Lisbon, Portugal; 2 INESC-ID, Instituto Superior Técnico, Universidade de Lisboa, Av. Rovisco Pais 1, 1900-001 Lisbon, Portugal

**Keywords:** Biclustering, Synthetic data generator, Time series analysis, Unsupervised learning

## Abstract

**Background:**

Biclustering is increasingly used in biomedical data analysis, recommendation tasks, and text mining domains, with hundreds of biclustering algorithms proposed. When assessing the performance of these algorithms, more than real datasets are required as they do not offer a solid ground truth. Synthetic data surpass this limitation by producing reference solutions to be compared with the found patterns. However, generating synthetic datasets is challenging since the generated data must ensure reproducibility, pattern representativity, and real data resemblance.

**Results:**

We propose G-Bic, a dataset generator conceived to produce synthetic benchmarks for the normative assessment of biclustering algorithms. Beyond expanding on aspects of pattern coherence, data quality, and positioning properties, it further handles specificities related to mixed-type datasets and time-series data.G-Bic has the flexibility to replicate real data regularities from diverse domains. We provide the default configurations to generate reproducible benchmarks to evaluate and compare diverse aspects of biclustering algorithms. Additionally, we discuss empirical strategies to simulate the properties of real data.

**Conclusion:**

G-Bic is a parametrizable generator for biclustering analysis, offering a solid means to assess biclustering solutions according to internal and external metrics robustly.

## Background

Unsupervised learning techniques such as clustering can find global patterns from multivariate observations based on the similarities found along all variables. However, biomedical patterns (including regulatory modules from multivariate omic data) are local, only correlated on a subset of attributes [1]. In this context, biclustering algorithms yield unique advantages. First, they can unravel local patterns, i.e., group observations meaningfully correlated on a subset of all attributes. Second, they place greater flexibility in the definition of different types of patterns. Third, biclusters can overlap, meaning that a subject, sample, or biological entity can belong to more than one bicluster [2, 3]. Table 1 lists prominent areas for biclustering applications. Biclustering has been pervasively applied to diverse domains, such as clinical and biological [4–6], gene expression data [7, 8], recommendation tasks [9, 10], text mining [11], and time series analysis [12, 13].

**Table 1:** Application scenarios for biclustering

Domain	Data	Illustrative biclusters
Biomedical	Clinical [14, 15]	Groups of patients whose health records are correlated
	Gene expression [5, 16]	Groups of genes involved in functional processes and pathways
	Biological networks [17, 18]	Modules of genes and proteins with local interaction
	Physiological [4, 12]	Modules of sliding partitions of the signal across a subset of stimuli responses
Other	Recommendation systems [9, 19]	Groups of users who share the same rating patterns and behavioral patterns
	Text mining [11, 20]	Groups of documents with similar sets of words or topics
	Climate data [21, 22]	Regions with similar meteorological patterns
	Social sciences [23, 24]	Regions with similar social vulnerabilities
	Resources utilization [13, 25]	Regions with similar water or energy consumption patterns

While there are studies that use real data to compare the capacities of biclustering algorithms, this type of data has significant challenges due to the non-existence of a solid ground truth (a set of known and well-characterized internal data structure, regularities, or patterns) that would enable assessing the accuracy, sensitivity, and precision of the existing methods. Therefore, real data analysis only evaluates the internal coherency of the retrieved bicluster, disregarding an evaluation of false discovery rates [12, 26].

Considering the limitations of real data, biclustering studies have relied on synthetic data to test and improve their algorithms, where ground truth is available, and used external metrics to compare the capacity of biclustering algorithms to retrieve planted biclusters [1, 27–30]. Currently, the approach followed by previous studies suffers from three issues. *First*, each study generates its synthetic datasets, which is not only a time-consuming task but also is generally biased towards the approach under evaluation [31]. *Second*, most comparison studies do not make the produced data available; synthetic datasets are still scarce and poorly characterized, making replicating the data with the same characteristics unfeasible. *Third*, available synthetic data lack expressivity concerning temporal dynamics, flexible structures, and real data resemblance.

We propose *G-Bic*, a synthetic data generator conceived to produce heterogeneous datasets for biclustering. Several options for pattern coherence, biclustering structure, and overlapping are available. In particular, G-Bic is the first contribution to generating multivariate data with numeric, symbolic, and time-series data; therefore, it conforms to diverse application domains. G-Bic is implemented as a Java GUI for generating datasets with usability guarantees and a programmatic API. In addition to the software description of the tool, a set of empirical principles are given to properties for dataset generation in several domains.

The article is organized as follows: this remaining section defines the biclustering task, the evaluation strategies for biclustering algorithms, and the related work regarding simulating synthetic data. Section Implementation discusses the features of the generator. Section Results presents baseline datasets for algorithm assessment and discusses empirical methodologies to generate synthetic similar to real data, illustrating the capacities of G-Bic to generate synthetic datasets and emulate real datasets. Finally, Conclusion draws concluding remarks and implications from this work.

### Biclustering

**Fig. 1:**
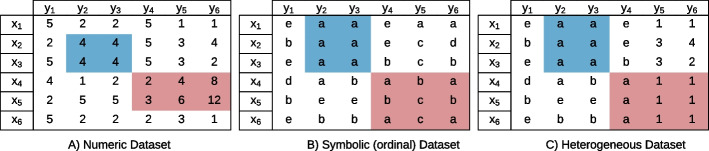
Example of a biclustering solution (made of two biclusters: blue and red) on three datasets: **A** numerical, **B** symbolic, and **C** heterogeneous

Given a two-dimensional dataset *A*, defined by *n* observations (rows) $X = \{x_1,..., x_n\}$ and *m* attributes (columns) $Y = \{y_1,...,y_m\}$, a **Bicluster** *B* corresponds to a subset of rows $I \subseteq X$ and columns $J \subseteq Y$ of the original matrix. We denote by $b_{ij}$ the value corresponding to row *i* and column *j* in the bicluster *B* [2]. A **Biclustering Solution** is a set of *q* biclusters discovered by an algorithm, $\{B^1,B^2,...,B^q\}$, that satisfy specific homogeneity and statistical significance criteria [2, 32]. Figure 1 shows biclustering solutions on numerical, symbolic, and heterogeneous datasets.

**Fig. 2:**
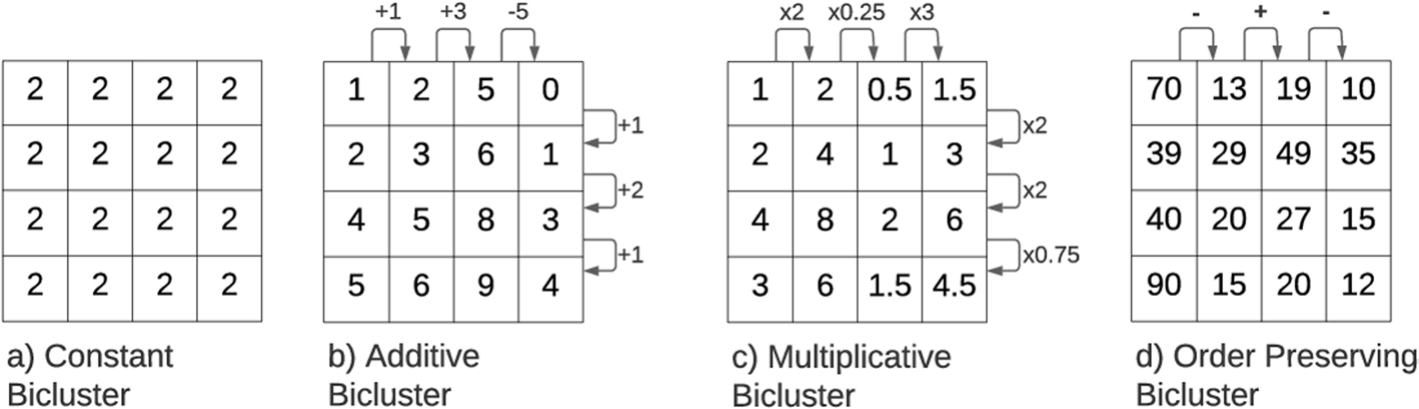
The coherency of a bicluster shows how the values inside the bicluster correlate with each other

The **coherence** identifies the correlations inside a bicluster [1]. We highlight four significant coherency types, illustrated in Fig. 2: *Constant*, assuming that the bicluster has constant values, *Additive*, assuming that the values inside the bicluster are explained by a sum of factors, *Multiplicative*, assumes that the values are explained by a product of factors, and *Order Preserving* if there is a permutation of the attributes such that the values show a linear ordering across the dimension.

**Fig. 3:**
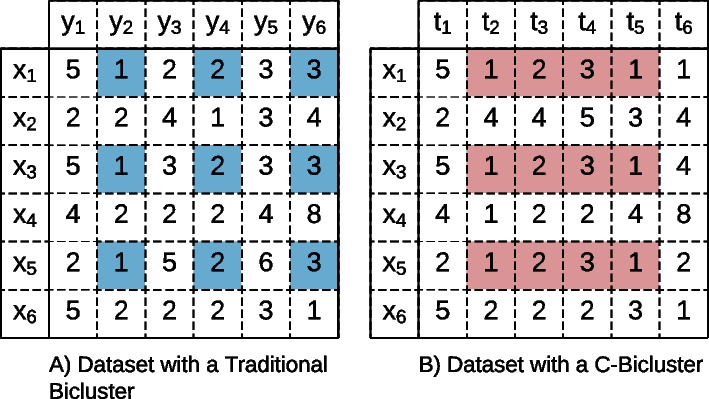
Diference between a **A** Traditional Bicluster and a **B** C-Bicluster. A C-Bicluster represents temporal patterns. Hence, placing column contiguity can be desirable

Usually, a biclustering algorithm does not consider the relative position between rows or columns. In time series data, columns correspond to time points, so the relative positioning between columns is relevant. Biclustering algorithms are classified as having temporal contiguity if they discover bicluster with contiguous columns, and the biclusters discovered by those algorithms are known as **C-Biclusters** [12, 13, 33, 34]. Figure 3 visually depicts the difference between a traditional bicluster and a C-Bicluster.

**Fig. 4:**
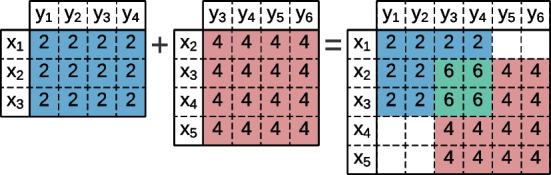
Plaid model with an **additive** cumulative function between two constant biclusters

The elements of a data matrix can be part of more than one bicluster. The contributions per biclusters to the data matrix are explained by plaid models [35]. Lazzeroni and Owen [36] proposed an *additive model* (Fig. 4) where the elements $a_{ij}$ of the data matrix are viewed as a sum of terms,

1 $$\begin{aligned} a_{ij} = \sum _{t=0}^{q}{\theta _{ijt}\rho _{it}\kappa _{jt}}, \end{aligned}$$

where $\theta _{ijt}$ defines a contribution for each bicluster *t*, and $\rho _{it}$ and $\kappa _{jt}$ are boolean values that state if the observation *i* and attribute *j* is present on the bicluster.

**Fig. 5:**
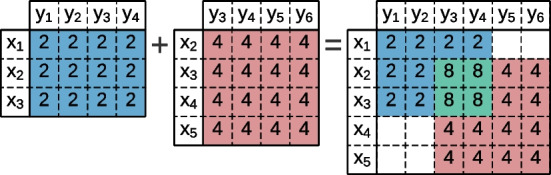
Plaid model with an **multiplicative** cumulative function between two constant biclusters

Madeira and Oliveira [2] proposed the *multiplicative model* (Fig. 5) that assumes that a product of bicluster layers generates the matrix values,

2 $$\begin{aligned} a_{ij} = \prod _{t=0}^{q}{\theta _{ijt}\rho _{it}\kappa _{jt}}. \end{aligned}$$

**Fig. 6:**
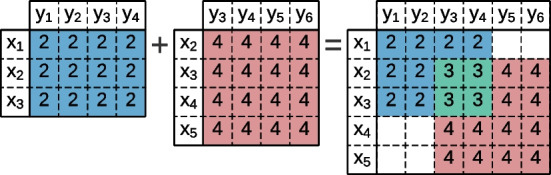
Plaid model with an **interpoled** cumulative function between two constant biclusters

Henriques and Madeira [37] introduced the *interpoled model* (Fig. 6) to better capture behaviors in alternative domains. In this model, each value is obtained by averaging the bicluster values.

3 $$\begin{aligned} a_{ij} = \frac{\sum _{t=0}^{q}{\theta _{ijt}\rho _{it}\kappa _{jt}}}{q}. \end{aligned}$$

### Evaluating biclustering algorithms

Evaluating biclustering correctness, homogeneity and statistical significance is challenging, as highlighted by a proliferation of different metrics [26, 38].

**External metrics**, also known as extrinsic or similarity evaluation metrics, are used when ground truth is known and quantify how well the biclustering algorithm recovers a planted/hidden biclustering solution [38]. The solutions obtained by biclustering algorithms are compared to the reference using a similarity measure based on variations of the F-Measure [39] or Jaccard scores [40]. These metrics are an essential tool to compare the correctness of the algorithms retrieving a biclustering and are used in studies developing new algorithms [5, 40] and in comparative studies [1, 27–29]. For a survey on external measures in biclustering, we refer to Horta and Campello [38].

**Internal metrics**, also known as quality metrics or coherence metrics, are used to evaluate the internal homogeneity of the bicluster [26]. Since biclustering has several types of patterns, defining homogeneity will depend on the assumed bicluster pattern. Different internal metrics are suited for different internal patterns. Examples are the *Variance*, formulated to detect constant biclusters, or the *Mean Squared Residue* focused on additive patterns [41, 42]. A few studies have recently used standardization techniques in the bicluster to develop flexible internal metrics to detect diverse patterns [26]. As indicated by Divina et al. [43], an advantage of this approach is to characterize their tendency. One way of doing it is to standardize data by row,

4 $$\begin{aligned} \hat{b}_{ij} = \frac{b_{ij}-\mu _{i}}{\sigma _{i}}, 1 \le i \le |I|, 1 \le j \le |J|, \end{aligned}$$

where $\mu _{i}$ is the mean value of row $x_i$ in the bicluster and $\sigma _{i}$ is the standard deviation of all the column values of row $x_i$. Let a virtual pattern be defined as:

5 $$\begin{aligned} \hat{\rho _j} = \frac{1}{\mid I \mid } \sum _{i=1}^{\mid I \mid }{\hat{b_{ij}}}. \end{aligned}$$

This pattern represents an average behavior of all observations in each attribute, and the virtual error (VE) [26, 43, 44] is thus defined as a difference between the row values and the virtual row,

6 $$\begin{aligned} VE(B) = \frac{1}{\mid I \mid \times \mid J \mid }\sum _{i=1}^{\mid I \mid }{\sum _{j=1}^{\mid J \mid }{\mid \hat{b}_{ij} - \hat{\rho }_{j} \mid }}, \end{aligned}$$

where $b_{ij}$ refers to the element (*i*, *j*) of the data subspace *B*, and |*I*|, |*J*| represent the total number of rows and columns. This metric is sensitive to constant, additive, and multiplicative patterns. Although order preserving patterns may fail to show a zero virtual error, significantly lower virtual errors for these subspaces are expected compared to non-homogeneous subspaces.

A more reduced set of intrinsic metrics has been proposed to evaluate the homogeneity of symbolic biclustering solutions. The symbolic biclusters are generally evaluated by assuming a symbolic pattern and measuring the noise as the percentage of elements in the bicluster that do not respect the pattern. Other strategies convert symbolic onto numeric patterns (using a traditional metric for numeric data) or consider metrics developed for clustering [45–47].

**Statistical significance metrics** evaluate how relevant the bicluster is in the data matrix compared to randomized data or null assumptions. These metrics are relevant since highly homogeneous biclusters can appear by chance, and these metrics reduce the occurrence of false positives. These metrics are used in previous application studies, often combined with internal metrics, to assess biclustering solutions [13].We refer to Henriques and Madeira [35] for a survey on statistical significance metrics.

### Related work

In contrast to real data (where no ground truth is given), synthetic datasets offer the advantage of evaluating biclustering algorithms based on the correctness of the retrieved biclustering solutions [38, 48]. Synthetic datasets are widely used in the biclustering literature to support algorithmic development [16, 40, 49, 50], the design of merit functions and evaluation metrics [26, 38], and algorithm comparison [27, 29].

Hochreiter et al. [40] compared their proposed algorithm in datasets generated by previous authors [16, 27] and custom-generated datasets. A biclustering generator was made available in the “fabia” R package; however, it uses the same additive generation model that the FABIA algorithm assumes to be true. The “isa2” R package also makes a synthetic data generator available, but it lacks diverse coherency options and only generates overlapping biclusters of equal size. Horta and Campello [38] generated seven synthetic data collections to evaluate external measures. These datasets were made with the intent of reproducing previous studies [2, 27, 29, 40]. Pontes et al. [26] evaluated experimentally internal metrics by generating synthetic datasets with different coherences and noise values.

Synthetic datasets are also used in most comparative studies, i.e., studies developed to systematically compare the performance of biclustering algorithms. Prelić et al. [27] generated biclustering datasets with constant and additive coherencies. Their objective was to compare biclustering algorithms given varying degrees of noise and overlap. This approach was later followed by Bozdağ et al. [28], and Padilha and Campello [30] by expanding the study variables, including the data size, the number, and shape of biclusters, and their possible coherencies.

Generating synthetic data is a time-consuming task due to the development and running of the generating scripts. Assumptions about the patterns in the data must be made, risking a bias toward the algorithm under evaluation. The generated data (and accompanying scripts) are often unavailable, raising reproducibility issues. Challenges with generating synthetic data lead to the development of independent data generators. Data generators developed in areas such as pattern mining [51] and clustering [52] are not helpful because they cannot simulate the pattern diversity in biclustering and the overlapping between biclusters. Therefore, there is a need for data generators specific to biclustering. It is also worth noticing that synthetic data for biclustering demands well-defined ground truth and parametrizable patterns. Although the use of real data simulators [53–55], or generative AI approaches [56, 57] can provide unique opportunities for simulating the properties of real data, they lack the flexibility to be applied in several domains, create a well-defined ground truth, and provide parameterization facilities within usable interfaces.

*BiBench* is a contribution capable of generating synthetic datasets [29]. This generator creates datasets with coherency patterns following definitions by Aguilar-Ruiz [44] and overlapping between biclusters given by the additive plaid model [36]. However, BiBench was limited in bicluster patterns: the generated datasets were numeric, all biclusters in the dataset followed the same type of pattern, and the patterns showed constant values across columns. This generator also lacked overlapping and noise options. Therefore, BiBench cannot generate data where the observations show different correlations between the attributes.

*BiGen*, later proposed by Henriques [58], fixed these limitations and broadened the set of features by allowing the generation of biclusters of both symbolic and numeric natures, with dynamic structures, considering all patterns, and with parameterizable overlapping and quality options. However, this data generator lacks background definitions, the generation of heterogeneous data, and temporal biclusters.

Developed for triclustering (an unsupervised task with similar data challenges as biclustering), *G-Tric* introduced new concepts such as generating (possibly distinct) patterns over more than one dimension, admitting that the background and the bicluster could follow different noise structures, and contiguity options that are useful to simulate time series data. Since G-Tric was developed for triclustering, the generated datasets are not directly applicable to biclustering. Additionally, compared to G-Bic, G-Tric lacks options to generate heterogeneous data.

## Implementation

We propose *G-Bic*, a fully parametrized generator of heterogeneous and temporal data focused on the necessities of biclustering. G-Bic is the first generator with options for (1) generating heterogeneous data (i.e., mixed variable types) with planted patterns; (2) handling temporal data, including contiguity assumptions on the time dimension; and (3) higher degrees of flexibility for parameterizing the coherence (type and strength), structure (number and size), and quality of the biclusters, surpassing the limitations of earlier generators. In particular, G-Bic:

1. Allows to define the pattern in both rows and columns;
2. Uses personalized quality options for the dataset background and the planted biclusters;
3. Extends the overlapping parametrization to allow defining the fraction of patterns that can overlap and their overlapping extent;
4. Generates either homogeneous or heterogeneous datasets, with non-identically distributed variables, combining symbolic and numeric variables;
5. Generates temporally contiguous biclusters.

**Fig. 7:**
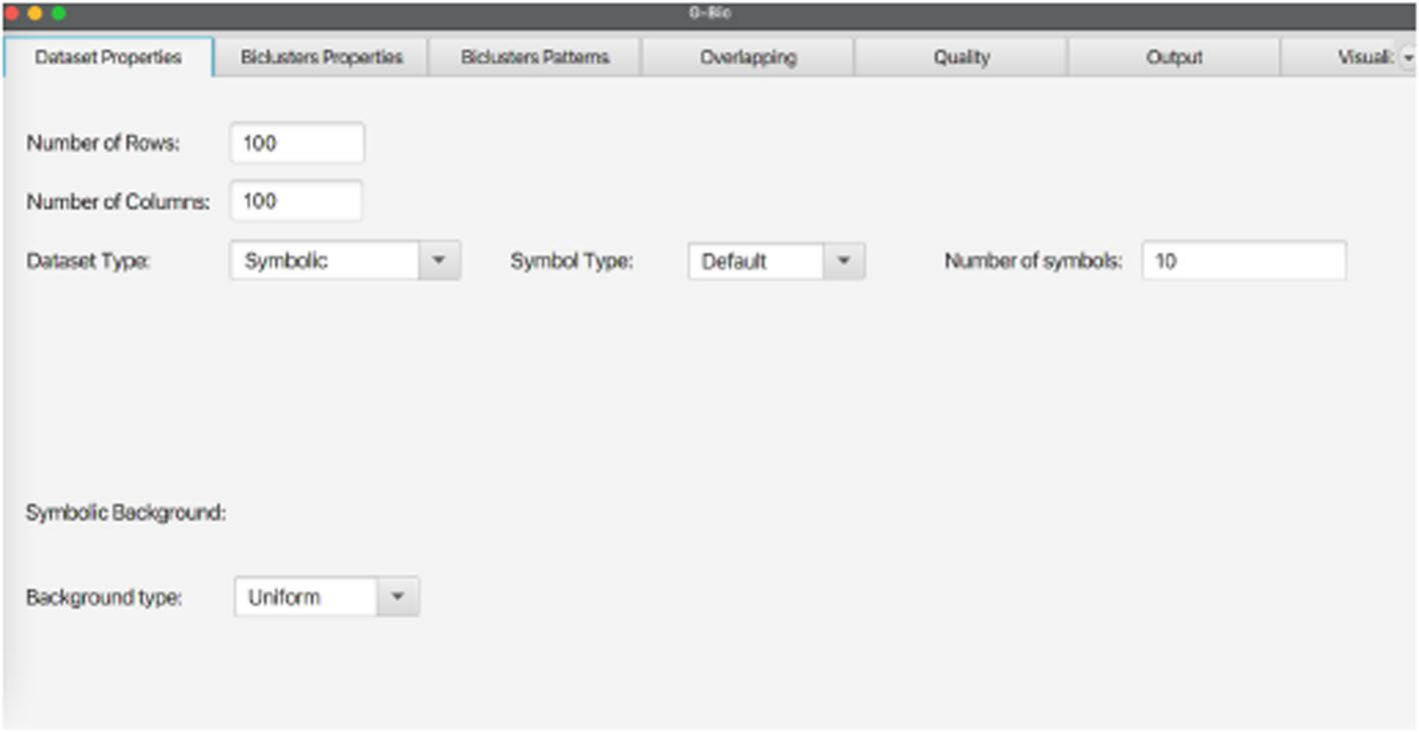
Overview of the GUI implementation, guaranteeing usability for data simulation. Accompanying the executable, the source code and a tutorial are available at https://github.com/jplobo1313/G-Bic

Furthermore, *G-Bic* is implemented as a GUI (Graphical User Interface), developed using JavaFX for easy usability and a programmatic API for additional control over the data simulation facilities. Figure 7 shows the GUI implementation. As supplementary material, a tutorial is made available for the guided use of G-Bic. In the remainder of this section, we describe the proposed generator. Each subsection describes a tab of the G-Bic GUI.

### Dataset properties

The initial step in dataset generation entails delineating its structure without a planted bicluster, an option available within the *Dataset Properties* tab. The structure is determined by specifying the dataset’s dimensions in terms of rows and columns, elucidating the attribute types present in the dataset (numeric, symbolic, or heterogeneous), and elucidating the statistical underpinnings of the dataset.

When considering heterogeneous data, the user controls the percentage of numeric attributes compared to symbolic ones. If the user selects numeric features, he controls the attributes, integers, or real-valued, and the maximum and minimum values. Symbolic attributes are best used when the features are represented by symbols (such as characters) or a general trend. The user can then select the number of symbols by defining a custom list of symbols or a default alphabet.

The background controls the possible distributions for the values in the dataset. G-Bic makes available four types of backgrounds:

- *Uniform*, assuming that each alphabet element has equal probability to occur;
- *Normal*, considering a gaussian distribution to generate the dataset;
- *Discrete*, which gives the control of the probabilities of each symbol to the user;
- *Missing*, that assumes a null background.

### Bicluster properties

The *Bicluster Properties* controls the general properties of the generated biclusters, essentially, the number of planted biclusters and their size. The number of rows and columns of the biclusters are variable and are defined by either a *Gaussian* or *Uniform* distribution. If the objective of G-Bic is to simulate biclusters for time series analysis, the *Contiguity* option can be controlled to generate C-Biclusters.

### Bicluster patterns

The third stage is the definition of patterns likely to be observed in terms of type and shape that will be planted on the dataset.

There are four types of coherency patterns for biclustering: *Constant*, *Additive*, *Multiplicative*, *Order Preserving*. In addition, G-Bic considers a fifth type, *None*, that represents the non-existence of coherency across one dimension. This last type is proper when the desired biclusters are only coherent across one dimension, i.e., the rows or the columns.

Compared to previous biclustering generators BiBench and BiGen, G-Bic allows defining the patterns in both dimensions, meaning that a bicluster can have a constant coherency over columns and additive over rows. The bicluster element $b_{ij}$ is a mathematical relationship between a seed value $\mu$, a row factor $\alpha _i$, and a column factor $\beta _j$ (except for the order preserving).

Due to incompatibilities related to the mathematical modeling of the patterns, not all patterns are compatible. Table 2 shows each coherency’s possible pattern configurations and mathematical expressions.

**Table 2:** G-Bic allows users to select different patterns over rows and columns

Coherency	Row pattern	Column pattern	Mathematical formula	Dataset type
Order preserving	Order Preserving	None	n/a	Numeric and symbolic
	None	Order preserving		
Constant	Constant	Constant	$b_{ij} = \mu$	Numeric and symbolic
	None	Constant	$b_{ij} = \alpha _i$	
	Constant	None	$b_{ij} = \beta _j$	
Additive	Additive	Additive	$b_{ij} = \mu + \alpha _i + \beta _j$	Numeric
	Constant	Additive	$b_{ij} = \mu + \beta _j$	
	Additive	Constant	$b_{ij} = \mu + \alpha _i$	
Multiplicative	Multiplicative	Multiplicative	$b_{ij} = \mu \times \alpha _i \times \beta _j$	Numeric
	Constant	Multiplicative	$b_{ij} = \mu \times \beta _j$	
	Multiplicative	Constant	$b_{ij} = \mu \times \alpha _i$	

**Fig. 8:**
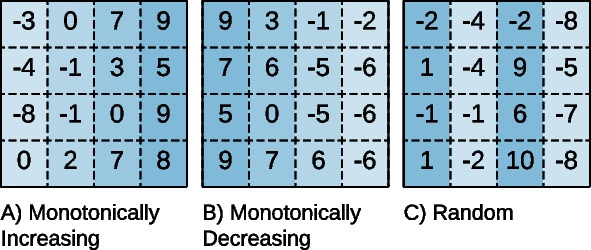
Considering the specific context of biclustering for time series and the Order Preserving coherency, G-Bic has three time profiles: **A** Monotonically Increasing, **B** Monotonically Decreasing or **C** Random

In the context of time series analysis and the generation of C-biclusters, if the coherency is chosen as order preserving, G-Bic allows the generation of temporal profiles that are either: *Monotonically Increasing*, *Monotically Decreasing*, or *Random*. These sub-coherencies, illustrated in Fig. 8, are exclusive for C-Biclusters, since the relative positioning of the columns is relevant.

### Overlapping

After generating the isolated biclusters, the next step is to define how they overlap.

G-Bic admits four possible *plaid coherencies*, i.e., how the values of overlapping biclusters add to the final value in the matrix: *additive* as defined in Eq. 1, *Multiplicative* as defined in Eq. 2, *Interpoled* as defined in Eq. 3 and, *None*, where the value in the datasets corresponds to the last generated bicluster.

The percentage of overlapping biclusters and the maximum number of interactions can be controlled. If the percentage is defined as $40\%$ and the maximum number of interactions is defined as 3, then $60\%$ of the biclusters will not overlap, and the ones that do overlap no more than 3 biclusters will overlap in a single matrix element. It is also possible to define the percentage of overlapping rows, columns, and elements between biclusters.

### Quality

The quality of the data and generated subspaces is parameterizable by assigning *missing*, *noise*, or *error* elements. The distinction between *noise* and *error* is done by a personalized *noise threshold* parameter, defining the tolerated deviation for the numerical values in the dataset and the biclusters. In G-Bic, noise is defined by fluctuations within the threshold, and errors are fluctuations beyond that.

### Output and visualization

After generating the biclustering solutions, G-Bic produces three files:

- **.json file**: Containing the settings that generated the dataset;
- **.txt file**: Listing the generated biclusters;
- **.tsv file**: Containing the dataset.

**Fig. 9:**
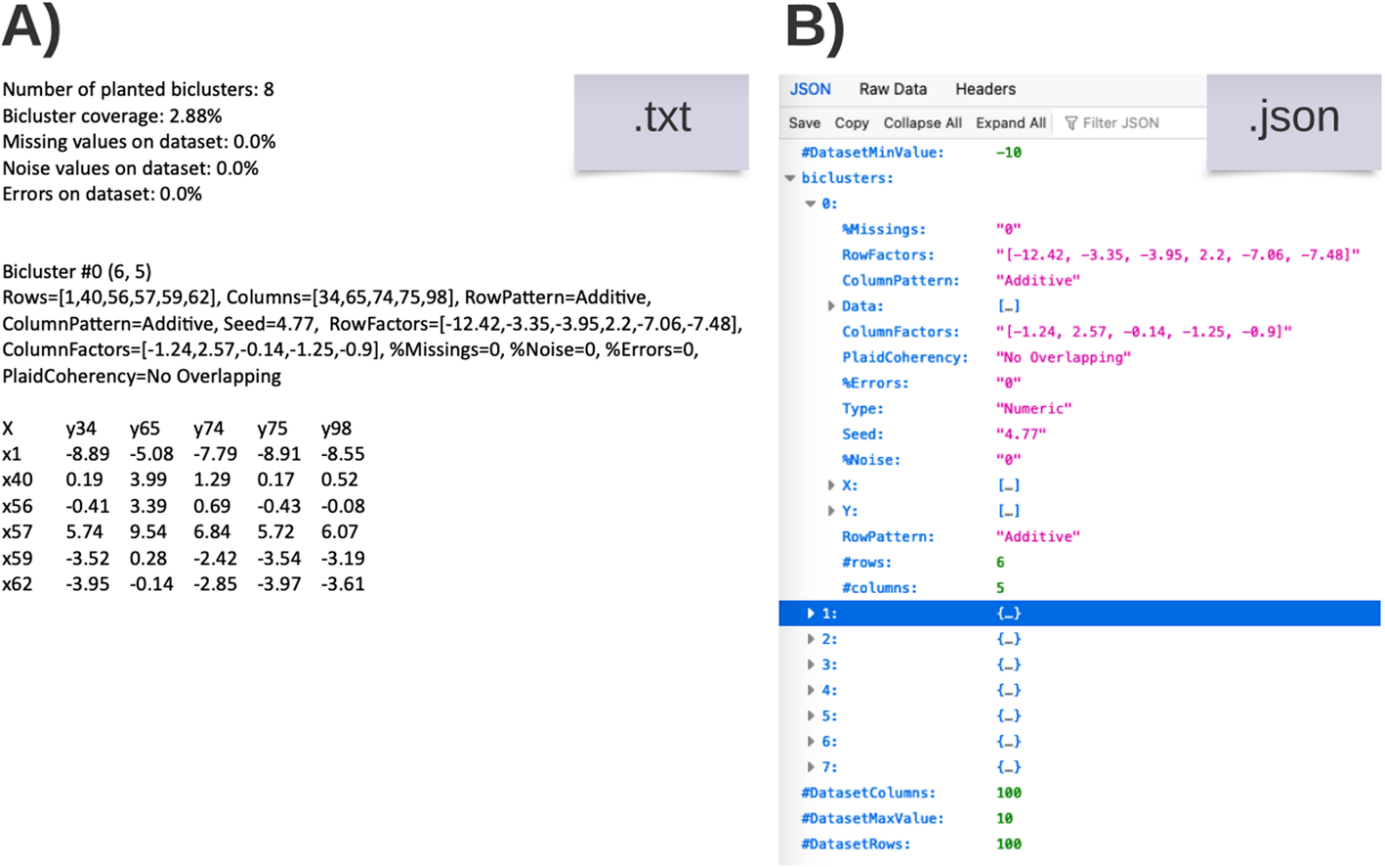
G-Bic generates files with a focus on accessibility. In **A**, the *.txt* generated by G-Bic has the information for each bicluster in a human-friendly view. In **B**, *.json* file has the information associated with the dataset and the biclusters structured to import the data into a programming script easily

The structure of the *.json* and *.txt* files are shown in Fig. 9. The three files make the results from G-Bic easily readable either by humans or automatically by any programming language with a *json* and *tsv* reader library. In addition to the files, G-Bic makes available a graphical tool to visualize the produced subspaces using heatmaps.

## Results

In this section, we provide default configurations to generate reproducible benchmarks to evaluate and compare the properties of biclustering algorithms. **First**, the simulation of simple synthetic datasets to evaluate the performance of biclustering algorithms by controlling essential parameters of G-Bic. We begin by producing three illustrative synthetic datasets. Then, we discuss baseline datasets and provide a guiding criterion for using G-Bic to establish experimental comparisons between biclustering algorithms. The **second** part of this section concerns the simulations of data resembling real data. We begin by discussing conclusions by authors in multiple biclustering domains and provide the settings to generate reference datasets for each application domain. Finally, we show an empirical approach in a scenario where no studies are available and how to use biclustering algorithms to infer properties for synthetic dataset generation. This empirical approach is illustrated with a gene expression microarray dataset.

### Illustrative datasets

**Fig. 10:**
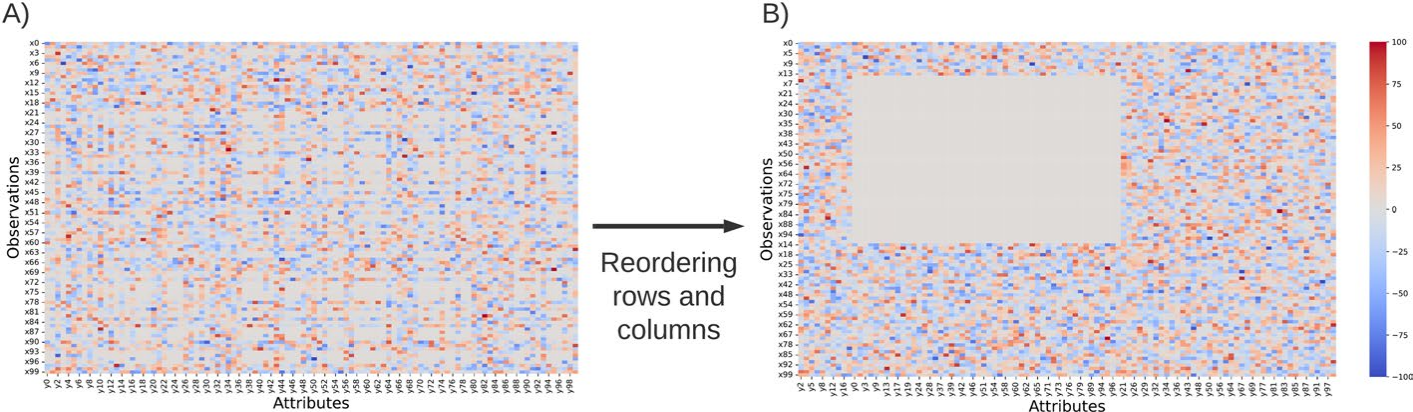
Synthetic dataset with a single constant bicluster. The produced dataset has the appearance of (**A**). For visualizing the biclustering solution, a reordering of rows and columns is needed to visualize the bicluster in (**B**)

**Fig. 11:**
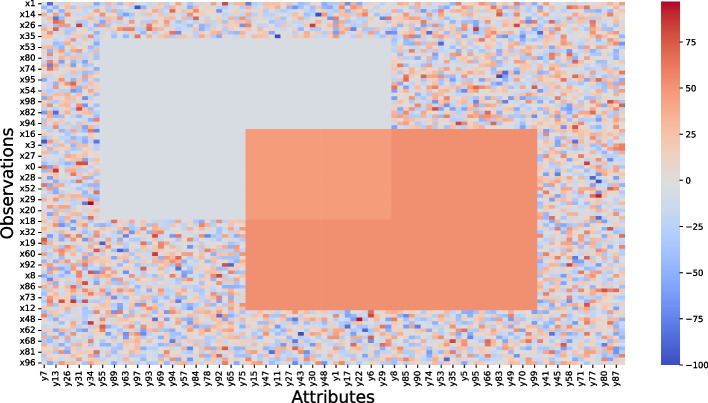
G-Bic generates biclusters with plaid assumptions, representing biological scenarios where genes participate in several biological processes

**Fig. 12:**
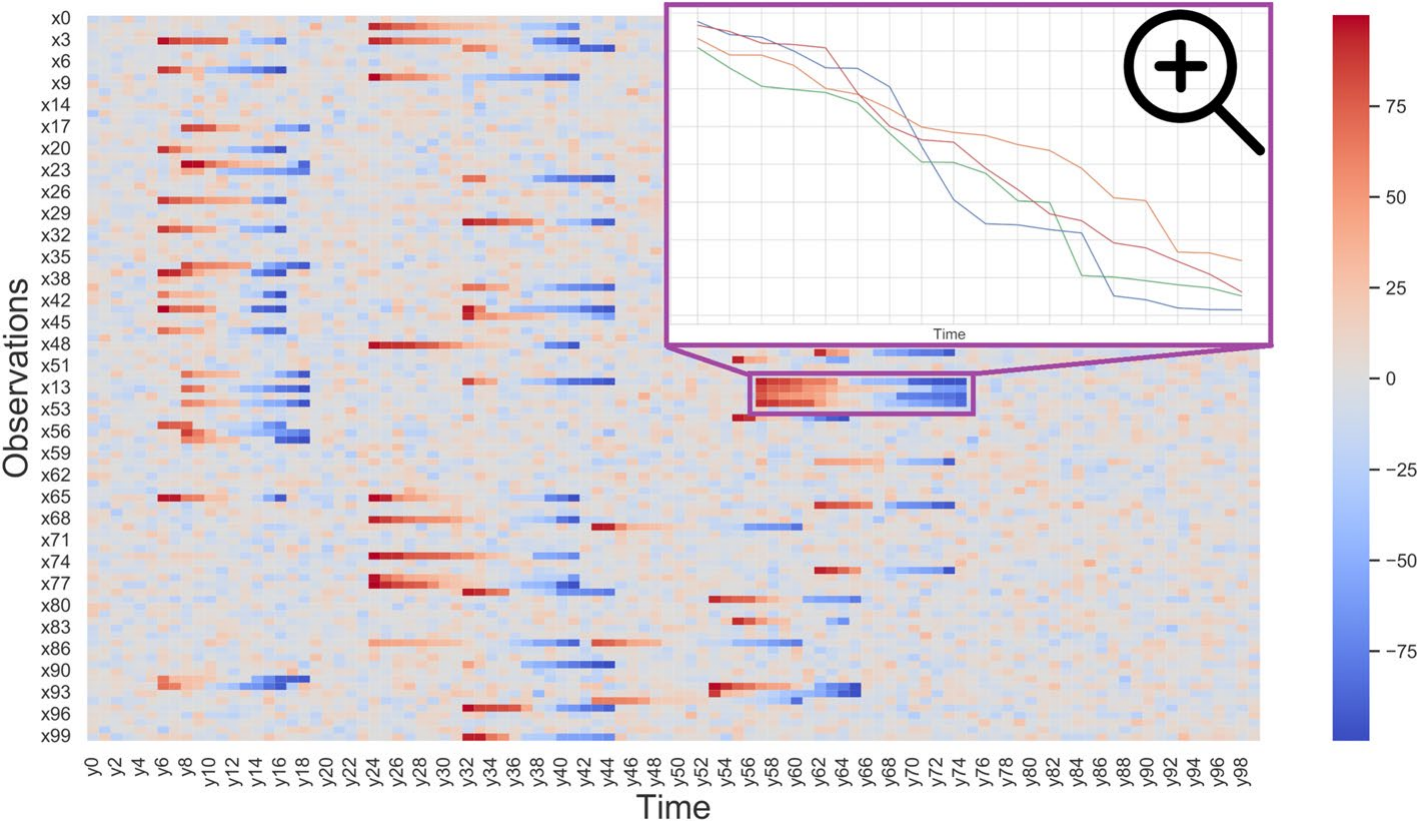
G-Bic generates biclusters representing temporal contiguity patterns. Parameters were selected to simplify the visualization. A close-up of one of the order-preserving biclusters shows four rows with a similar pattern over a few contiguous time points

In this section, we illustrate the capacities of G-Bic to simulate synthetic datasets for comparative studies. Assessing biclustering algorithms in simple solutions is an essential step to characterize the behavior of the algorithms and identify their limitations [1, 27–30]. The settings for these datasets are in Table 3. We consider three scenarios: in the *first* scenario, a synthetic dataset with a single bicluster, illustrated in Fig. 10. *Second*, we illustrate the plaid models by illustrating a dataset with an overlapping between two biclusters, illustrated in Fig. 11. *Finally*, the last illustrative dataset shows the temporal patterns created by G-Bic, illustrated in Fig. 12.

**Table 3:** Setting for the illustrative datasets

Properties		Illustrative 1	Illustrative 2	Illustrative 3
Dataset	Data type	Real-valued		
	Size	$100 \times 100$		
	Alphabet	[-100,100]		
	Background	N(0,30)		
Biclusters	Number	1	2	10
	Number of rows	50	50	U(3,12)
	Number of cols	50	50	U(10,20)
	Row Pattern	Constant	Constant	None
	Col Pattern	Constant	Constant	Order Preserving
	Contiguity	No	No	Yes
	Time Profile	None	None	Monotonically Decreasing
Overlapping	Plaid Coherency	No Overlapping	Additive	No Overlapping
	% of Overlapping biclusters	N.A	100 %	N.A
	Maximum number of bicluster interations	N.A	2	N.A
	% of overlapping elements	N.A	50 %	N.A
	% of overlapping rows	N.A	50 %	N.A
	% of overlapping columns	N.A	50 %	N.A
	Quality	No noise, errors or missing values		

### Baseline datasets

Six data domains were considered, each varying with regards to variable types (**R**-Real Valued, **S**-Symbolic, **B**-Binary, **I**-Integer, **C**-contiguous, and **H**-Heterogeneous), represented in Table 4. While these datasets do not help compare biclustering algorithms, they serve as a reference baseline to generate comparable, reproducible dataset benchmarks.

**Table 4:** Setting for baseline datasets

	Properties	Dataset R	Dataset S	DatasetB	Dataset I	Dataset C	Dataset H
dataset	Data type	Real-valued	Symbolic	Integer	Integer	Real-valued	Heterogeneous (50% real, 50% symbolic)
	Size	$1000 \times 100$	$1000 \times 100$	$1000 \times 100$	$1000 \times 100$	$1000 \times 100$	$1000 \times 100$
	Alphabet	[-100,100]	{a,b,c,d,e}	{1,0}	[0,100]	[-100,100]	[-100,100] and {a,b,c,d,e}
	Background	N(0,30)	Missing	Missing	Uniform	Uniform	Missing
bics	Number	1	1	1	1	1	1
	Dimensions	$50 \times 50$	$50 \times 50$	$50 \times 50$	$50 \times 50$	$50 \times 50$	$50 \times 50$
	Contiguity	No	No	No	No	Yes	No

The most studied parameter in biclustering algorithms is their robustness to the presence of noise in numeric datasets [1, 27, 29, 30]. G-Bic has three different parameters to control the quality of the datasets: the number of missing values, the noise (small fluctuations of the dataset) and the number of errors in the dataset (quantity of significant fluctuations of the dataset).

Another dataset characteristic often studied is the overlap between bicluster solutions. This is challenging due to the inherent restrictions associated with some biclustering searches and the complexity of plaid models [59]. Previous studies compare the performance of biclustering algorithms in overlapping biclusters by varying the row and column overlap degree [27, 29, 30]. G-Bic allows not only to personalize the percentage of overlapping elements over rows and columns but also to define how many biclusters will overlap and the type of plaid coherency (additive, multiplicative, and interpoled).

### Simulating datasets based on a literature review

In this section, we begin discussing the simulation of data similar to real data. To ensure the coverage of different data domains, we focus on five popular areas of application of biclustering: gene expression data, text mining, recommendation systems, biomedical, and Spatio-temporal data. We choose one reference dataset for each applicational domain based on a previous biclustering study, summarized in Table 5 , and explained below.

**Table 5:** Example datasets considered for each simulation scenario

	Dataset Context	Description	Dimensions	Size
1	Gene Expression	Arabidopsis [60]	Genes $\times$ Conditions	$21031 \times 351$
2	Recommendation Systems	MovieLens-20M [19, 61]	Users $\times$ Movies	$138000 \times 27000$
3	Text Mining	Reuters-21578 [62]	Terms $\times$ Documents	$29930\times 21578$
4	Clinical Data	PMSI2013 [6]	Patients $\times$ Clinical Data	$49231\times 7941$
5	Spatio-Temporal data	fMRI time series [12]	Brain Regions $\times$ Time	$30 \times 150$

In the context of **gene expression** data, it is worth noticing that transcriptomic data is a prevalent application domain for biclustering, so studies often consider RNAseq or microarray data when comparing the performance of their algorithms by using both internal and biological relevance metrics [38]. Comparative studies [1, 27–29] and applications reviews [7, 32] highlight this observation. From these studies, we choose the arabidosis microarray data that captures the expression levels of a few thousand genes on a few hundred experimental conditions [60] , hence representative of the dimensionality and data size of transcriptomic data.

For **recommendation systems**, we considered the MovieLens-20 M dataset, a popular benchmark dataset in several biclustering studies. Recommendations system datasets use integers to represent the preferences of thousands of users over thousands of products, frequently associated with a high sparsity [19, 61, 63–65].

In **text mining**, Reuters-21578 is used in most biclustering studies and consists of thousands of terms over thousands of documents, with a high sparsity [62, 66].

The fourth dataset considers **Clinical data**. While clinical data is often used in biclustering studies, their general characteristics vary from application to application. Our primary reference is the study by Vandromme et al. [6]. In this context, the reference clinical dataset has a heterogeneous nature, high sparsity, and hundreds of observations and attributes.

The final simulated dataset comes from **Spatio-temporal data**. Spatio-temporal data has two challenges: First, since the application domains, so do the data characteristics. Second, most studies do not share the data or its specific characteristics. We considered the work by Castanho et al. [12], who evaluated the capacities of biclustering on fMRI data.

Considering the settings based on the definitions from Madeira and Oliveira [2] and the conclusions by studies that apply biclustering algorithms in each domain, the parameters for the simulated datasets are present in Table 6.

**Table 6:** Settings to simulate real datasets

Parameters		Gene expression	Recommendation systems	Text mining	Clinical data	Spatio-temporal data
Dataset properties	Number of rows	10000	200000	30000	50000	30
	Number of columns	100	30000	20000	8000	150
	Heterogeneous?	No	No	No	Yes	No
	Properties	Background with 10% noise	Background with 95,5% Missing values	Background with 99,8% missing values	Background with 99,8% missing values	Background with 50% noise and 20% errors
Bicluster properties	Number of biclusters	500	3000	70	30	20
	Rows structure	U(80,400)	U(30,70)	U(1000,10000)	U(20,100)	U(2,4)
	Columns structure	U(20,40)	U(3,7)	U(600,6000)	U(5,15)	U(7,10)
	Contiguity	No	No	No	No	Yes
Biclustering patterns		Additive and Order Preserving	Order Preserving	Constant and Order Preserving	Order Preserving	Additive and Multiplicative
Overlapping		10% bics with additive overlap	None	None	None	None

### A methodology to simulate real data

**Fig. 13:**
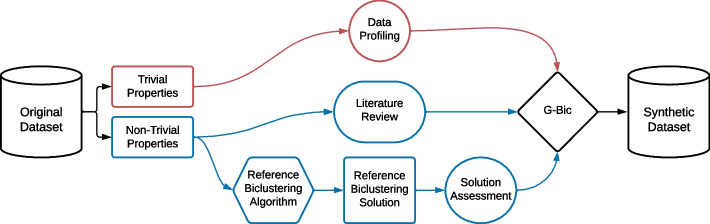
Methodology to simulate synthetic data resembling real data. A reference biclustering algorithm produces results under different homogeneity criteria, from which synthetic datasets are produced

In the previous section, we discussed the parameterizations to generate datasets based on previous research. This section proposed an empirical methodology to decide on the G-Bic parameters for dataset simulation. **First**, we explain the general methodology, which is illustrated in Fig. 13. **Second**, we illustrate this methodology by applying it to a gene expression dataset.

**Fig. 14:**
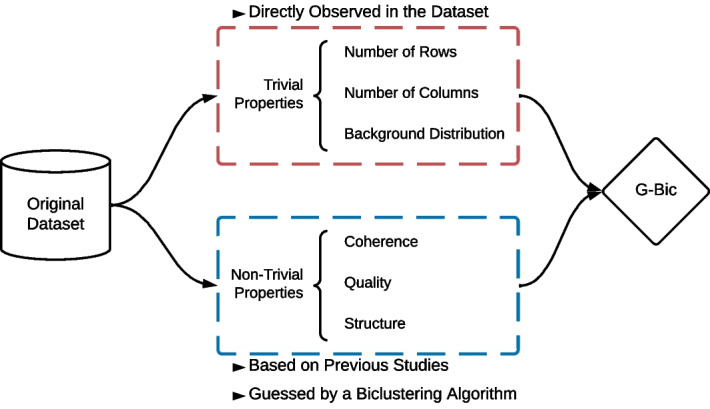
The datasets have trivial properties that can be directly observed in the dataset and non-trivial properties. To determine these non-trivial properties, we suggest an extrapolation based on previous studies on the application domains or using a biclustering algorithm as a reference

In this methodology, we consider a reference dataset. Any dataset has two types of properties, illustrated in Fig. 14: Trivial and Non-trivial properties. The trivial properties can be directly observed in the dataset. Examples of trivial properties are the dataset size, the background distribution, or the percentage of missing values. However, there are a few non-trivial properties, such as the coherence assumption in the data and the number and structure of the biclusters. For these, we propose that the parameters should be either decided from: (**A**) the properties of the biclustering solutions on previous studies on the application domain or (**B**) a reference biclustering algorithm as a reference to identify the properties of the patterns in the original data.

This methodology has the challenge of being dependent on the choice and parameterization of a reference biclustering algorithm. While several biclustering algorithms could be used to infer the dataset’s properties, we suggest the use of BicPAMS since it is a flexible, highly parametrizable algorithm with well-defined solutions in terms of coherency patterns, integrating state-of-the-art contributions on pattern-based biclustering [5, 37].

**Fig. 15:**
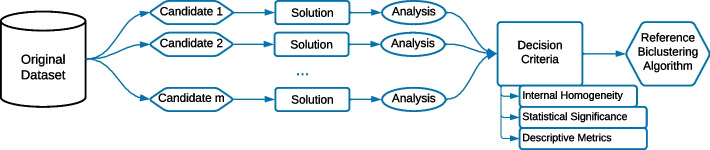
To guarantee that the biclusters represent patterns in the original data, several candidates could be used with criteria for decision-making

A potential issue with using a biclustering algorithm as a reference to extract relevant features for the synthetic data generation is the risk of generating biased results towards the specific algorithm. To guarantee that the reference biclustering solution is representative of the patterns in the original dataset, we propose that several reference biclustering candidates should be used, either various biclustering algorithms or different parametrizations of the same algorithm, as illustrated in Fig. 15. These candidates should be tested and evaluated with decision criteria, which could consist of either (**A**) internal metrics, selecting the candidate that produced the most homogeneous solutions given an adequate metric, (**B**) statistical significance metrics, selecting the candidate that produced the most statistically relevant biclustering solution or (**C**) descriptive metrics such as the number of biclusters or their size as decisive criteria.

#### Illustrative results

**Fig. 16:**
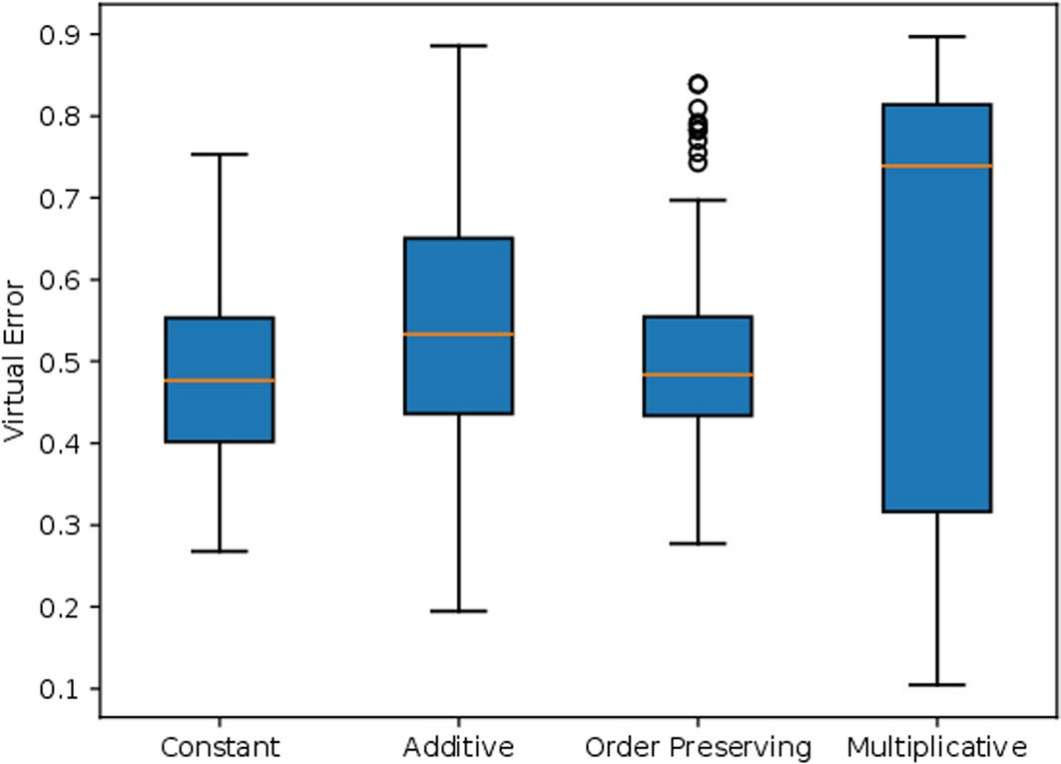
Virtual Error of each reference candidate algorithm. Each candidate generates a biclustering solution with a different homogeneity. The “constant” candidate creates solutions with lower virtual error

To illustrate the approach, we considered the simulation of synthetic data resembling the *dlblc dataset*, which can be accessed in https://web.ist.utl.pt/rmch/bicpams/. The dlblc dataset measures the expression of 660 genes across 180 experimental conditions [37, 67]. To decide the coherence of the biclustering solution, we considered four iterations of BicPAMS, considering four coherencies: Constant, Additive, Multiplicative, and Order Preserving. Table 7 describes the relevant parameters used in BicPAMS. We considered internal homogeneity a decision criterion evaluated by the virtual error metric. Figure 16 shows the virtual error for each configuration, and Table 8 shows the size of each biclustering algorithm. Based on the homogeneity decision, we generate data with constant biclusters. We considered results from the previous section (overlapping and quality) for the additional parameters. Table 9 shows the G-Bic configurations that generate the synthetic dataset.

**Fig. 17:**
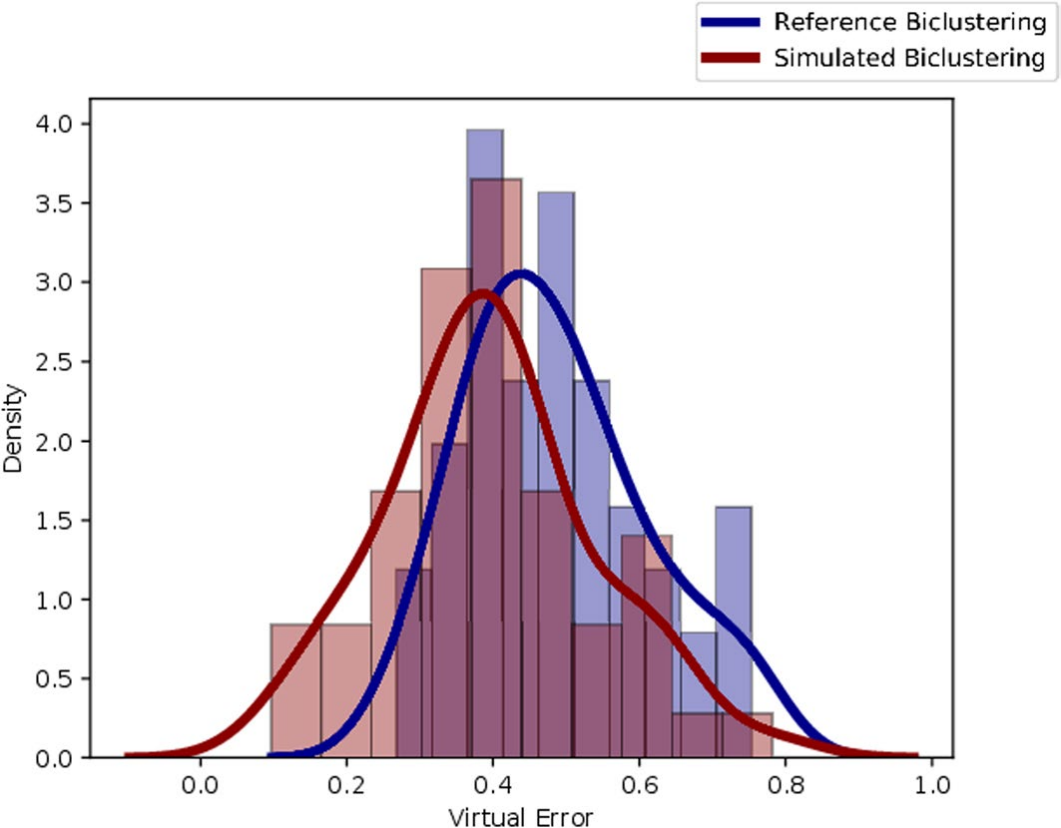
Distribution of the virtual error. The two biclustering solutions have a similar distribution in terms of internal quality, an indicator that the dataset produced by G-Bic potentially resembles the original data

Figure 17 compares the homogeneity distributions of the reference biclustering (which we assume to be representative of the original data) and the simulated biclustering (produced by G-Bic). We can observe that the distributions are similar.

**Table 7:** Parametrization of BicPAMS

Parameters	Values
Coherency strength	6
Merging overlap	80%
Pattern orientation	Rows
Minimum columns	4

We applied BicPAMS to the reference dataset *dlblc* based on the previously discussed methodology. The table only has the relevant parameters; the remaining were set as default

**Table 8:** The size of each BicPAMS candidate solution

Configuration (coherency)	Number of biclusters	Number of rows (average)	Number of columns (average)
Constant	52	73	4
Additive	7702	51	4
Order preserving	129	88	4
Multiplicative	1134	30	4

**Table 9:** Parametrization of G-Bic

Parameters		Values
Dataset properties	Number of rows	660
	Number of columns	180
	Dataset type	Numeric
	Data type of rows	Real valued
	Min value	$-100$
	Max value	100
	Background type	Normal (0,45)
Bicluster properties	Number of biclusters	52
	Row distribution	Normal (73,3)
	Number of columns	4
	Contiguity	None
Bicluster patterns	Row Pattern	Constant
	Column pattern	None
Overlapping settings	Plaid Coherency	Additive
	% of overlapping biclusters	10
	Maximum number of bicluster interactions	5
	% of overlapping rows	10
	% of overlapping rows	10
Quality	% Missing values on background	0
	% Missing values on biclusters	0
	% Noise values on background	0
	% Noise values on background	40
	Noise deviation	90
	% Errors values on background	0
	% Errors values on background	10

We used G-Bic to generate a synthetic dataset with similar properties based on the reference biclustering

## Conclusion

Biclustering algorithms, initially developed for gene expression data analysis, are gaining increased importance in several domains, which raises challenges regarding intrinsic data diversity. Since every developed algorithm tries to improve the state-of-the-art, reproducible synthetic benchmarks for a fair algorithmic comparison are increasingly important.

The main contribution of this study is the proposal of G-Bic, a synthetic data generator for biclustering. G-Bic provides a programmatic and graphical facility for the functional and standardized assessment of existing and upcoming unsupervised learning contributions in the field. Biclustering algorithms expand upon traditional clustering due to their flexibility in detecting patterns. G-Bic allows the generation of datasets with diverse characteristics, namely varying coherency and overlapping options. It is also the first contribution providing the ability to generate heterogeneous and time series data with planted pattern solutions, accompanying the expanding application scenarios for biclustering.

Complementarily, we provide benchmark datasets for a normative assessment of the characteristics of biclustering algorithms. Parameters to simulate real datasets are provided based on previous literature, and an empirical methodology to generate datasets similar to real data is given. These indications are expected to improve the current status on the evaluation of pattern mining and subspace clustering algorithms.

## Data Availability

The datasets generated during and/or analyzed during the current study are available in the G-Bic repository, https://github.com/jplobo1313/G-Bic. Availability and requirements: Project name: G-Bic. Project home page: https://github.com/jplobo1313/G-Bic. Operating system(s): Platform Independent. Programming language: Java. Other requirements: Java 16 or above. License: GNU GPLv3. ny restrictions to use by non-academics: No.
