# Correction: G-bic: generating synthetic benchmarks for biclustering

**DOI:** 10.1186/s12859-023-05628-y

**Published:** 2024-01-11

**Authors:** Eduardo N. Castanho, João P. Lobo, Rui Henriques, Sara C. Madeira

**Affiliations:** 1 LASIGE, Faculdade de Ciências, Universidade de Lisboa, Campo Grande 016, 1749-016 Lisboa, Portugal; 2 INESC-ID, Instituto Superior Técnico, Universidade de Lisboa, Av. Rovisco Pais 1, 1900-001 Lisboa, Portugal

## Correction: Castanho et al. *BMC Bioinformatics* (2023) 24:457 https://doi.org/10.1186/s12859-023-05587-4

Following the publication of the original article [1], the authors identified that the funding note was incorrect. The correct funding note is given below.

The incorrect funding is:

Funding was provided by Fundação para a Ciência e a Tecnologia (Grant Nos. 2021.07810.BD, DSAIPA/AI/0033/2019), H2020-RIA (Grant No. 818290), INESC-ID Research Unit (Grant No. UIDB/50021/2020), LASIGE Research Unit (Grant No. UIDB/00408/2020).

The correct funding is:

Funding was provided by Fundação para a Ciência e a Tecnologia (Grant Nos. 2021.07810.BD, DSAIPA/AI/0033/2019 and PTDC/CCI-CIF/4613/2020), H2020-RIA (Grant No. 818290), INESC-ID Research Unit (Grant No. UIDB/50021/2020), LASIGE Research Unit (Grant No. UIDB/00408/2020 and UIDP/00408/2020).

The original article [1] has been corrected.
